# Highly sensitive absorbance measurement using droplet microfluidics integrated with an oil extraction and long pathlength detection flow cell

**DOI:** 10.3389/fchem.2024.1394388

**Published:** 2024-05-13

**Authors:** Bingyuan Lu, James Lunn, Adrian M. Nightingale, Xize Niu

**Affiliations:** Mechanical Engineering, Faculty of Engineering and Physical Sciences, University of Southampton, Southampton, United Kingdom

**Keywords:** droplet microfluidics, oil extraction, long pathlength, absorbance, phosphate

## Abstract

In droplet microfluidics, UV-Vis absorption spectroscopy along with colorimetric assays have been widely used for chemical and biochemical analysis. However, the sensitivity of the measurement can be limited by the short optical pathlength. Here we report a novel design to enhance the sensitivity by removing oil and converting the droplets into a single-phase aqueous flow, which can be measured within a U-shape channel with long optical pathlength. The flow cells were fabricated via 3D printing. The calibration results have demonstrated complete oil removal and effective optical pathlengths similar to the designed channel lengths (from 5 to 20 mm). The flow cell was further employed in a droplet microfluidic-based phosphate sensing system. The measured phosphate levels displayed excellent consistency with data obtained from traditional UV spectroscopy analysis. This flow cell design overcomes the limitations of short optical pathlengths in droplet microfluidics and has the potential to be used for *in situ* and continuous monitoring.

## 1 Introduction

Over the last two decades, droplet microfluidics has been developed into a powerful tool for miniaturised chemical and biological assay and analysis (Baccouche et al., 2017; Cedillo-Alcantar et al., 2019; Schroen et al., 2021) due to its distinct advantages over conventional single-phase microfluidics. In droplet microfluidics, discrete droplets are formed by two immiscible fluids and each single droplet acts as an individual biochemical reactor (Shang et al., 2017). Chemicals mix rapidly in the droplet due to chaotic advection (Gan et al., 2023; Zhang et al., 2023), while mixing in the laminar flow of single-phase microfluidics is mainly achieved by relatively slow diffusion process (Ulkir et al., 2021). The droplet flow also reduces Taylor dispersion that is present in the single-phase flows (Hassan et al., 2019; Elvira et al., 2022). Therefore, droplet microfluidics enables less cross-contamination between samples and faster response for biochemical analysis, in addition to reduced reagent consumptions (Basova and Foret, 2015; Sun et al., 2020). By manipulating the two immiscible flows, high frequency droplet generation can be achieved for high throughput screening of targeted analytes or material synthesis (Sesen et al., 2017; Gelin et al., 2020; Payne et al., 2020).

One of the most common techniques used for droplet analysis is UV-Vis absorption spectroscopy (Trivedi et al., 2010; Hengoju et al., 2022), which enables the miniaturization of many well developed and calibrated laboratory assays into droplets and even for label-free quantification of analytes. However, due to the short optical pathlength across microscale microfluidic channels (normally below 1 mm) (Yang et al., 2017), absorbance measurement of droplets suffers from low sensitivity compared with benchtop spectrometers with 10 mm pathlength. This sensitivity limitation hinders the use of droplet microfluidics in applications where low detection limits are required to sense trace biochemical analytes.

In continuous microfluidics, several methods have been explored to enhance the detection sensitivity, including specific channel geometries [e.g., U/Z-shape channel (Ottevaere et al., 2015; Yang et al., 2017)], multi-pass cell (Llobera et al., 2007; Owens and Argueta-Diaz, 2022) and cavity-enhanced optics (Billot et al., 2008; Rushworth et al., 2015; Teggert et al., 2021). Some of those approaches were also demonstrated in droplet microfluidics. Yang et al. (2017) have utilised Z-shaped channels to “stretch” the droplet for accurate measurement of absorbance within individual pL volume droplets. Our group explored increased optical pathlength (Nightingale et al., 2020) using microfluidic chips made of Dyneon material—a hydrophobic fluoropolymer that becomes transparent after fabrication. However, the pathlengths cannot be extended arbitrarily since extending a droplet to a long liquid thread can lead to Rayleigh-Plateau instability (Baroud et al., 2010) and followed droplet break-up. The absorbance signals of droplets passing the Z-shape channels tend to be very noisy (Yang et al., 2017) caused by lensing effects from the head and tail hemispheres of the droplets in the elongated channel. Additionally, when a droplet passes through a Z-shaped channel, the curved droplet/carrier interface at the corners may cause further lensing effect and thus potential interference with the light path and transmitted light signals (Nightingale et al., 2020). Cavity enhanced spectroscopy (Neil et al., 2011; Rushworth et al., 2012) can increase the optical pathlength by incorporating an optical resonant cavity between two plano-concave mirrors, allowing multiple interactions between light and the targeted droplet. But its instrumental setup normally requires powerful light source, accurate alignment of advanced optics (e.g., highly reflective mirror, optical fibres) and detectors. The associated high cost and power consumption are not ideal for most of the portable/wearable sensors. Hence there is still a strong need to develop sensitive detection flow cells with low power consumption and affordable components.

In this paper, we present an alternative approach to enhance the sensitivity of absorbance measurements in droplet microfluidics. This technique involves the removal of the carrier oil via a PTFE membrane close to the detection point after achieving uniform mixing and reactions within the droplets. This oil-removal process transforms the droplets into a single-phase continuous flow, which can be precisely measured using integrated optical detection methods that benefit from an extended light pathlength. This approach combines the rapid mixing and reduced Taylor dispersion of droplet microfluidics with the enhanced sensitivity and long pathlength of continuous microfluidics. We designed flow cells with varying pathlengths and conducted characterization studies to demonstrate the diminished Taylor dispersion and improved detection sensitivity. Furthermore, we applied this concept to successfully quantify low-concentration phosphate in river water samples using a colorimetric assay, showcasing its practical applications.

## 2 Experimental section

### 2.1 Chemicals

All chemicals were purchased from Sigma Aldrich, United Kingdom unless otherwise stated. Ultrapure grade water was produced from 18.2 MΩ Barnstead EASYpure RODI for sample/reagent preparation. IR-820 dye (λ_max_ = 820 nm) was used to characterise the effective pathlength of the designed flow cell. A serial dilution of phosphate standard solutions from 2.5 to 10 uM were prepared from potassium phosphate monobasic for calibration. Fluorinert FC 40 oil (Acota Ltd., United Kingdom) was used as the continuous phase in droplet microfluidics. River water samples in this study were collected from River Itchen (United Kingdom) at high tide on the same day.

A modified phosphomolybdenum blue (PMB) assay was applied for phosphate quantification (Nagul et al., 2015). Two reagents are used, reagent A contains a mixture of 1.042 mL of 4 wt% ammonium molybdate tetrahydrate, 0.395 mL of 0.5 wt% potassium antimony tartrate hydrate, 3 mL 2.5 M sulphuric acid and 5.564 mL water; reagent B contains 0.1 wt% ascorbic acid aqueous solution. The PMB reagents and samples were introduced into droplet microfluidics at 1:1:3 volume ratios to perform the reaction. The assay involved the formation of phosphomolybdic acid followed by its reduction into a blue-coloured product phosphomolybdenum with a maximum absorption wavelength of 876 nm.

### 2.2 Detection flow cell and chip fabrication

**FIGURE 1:**
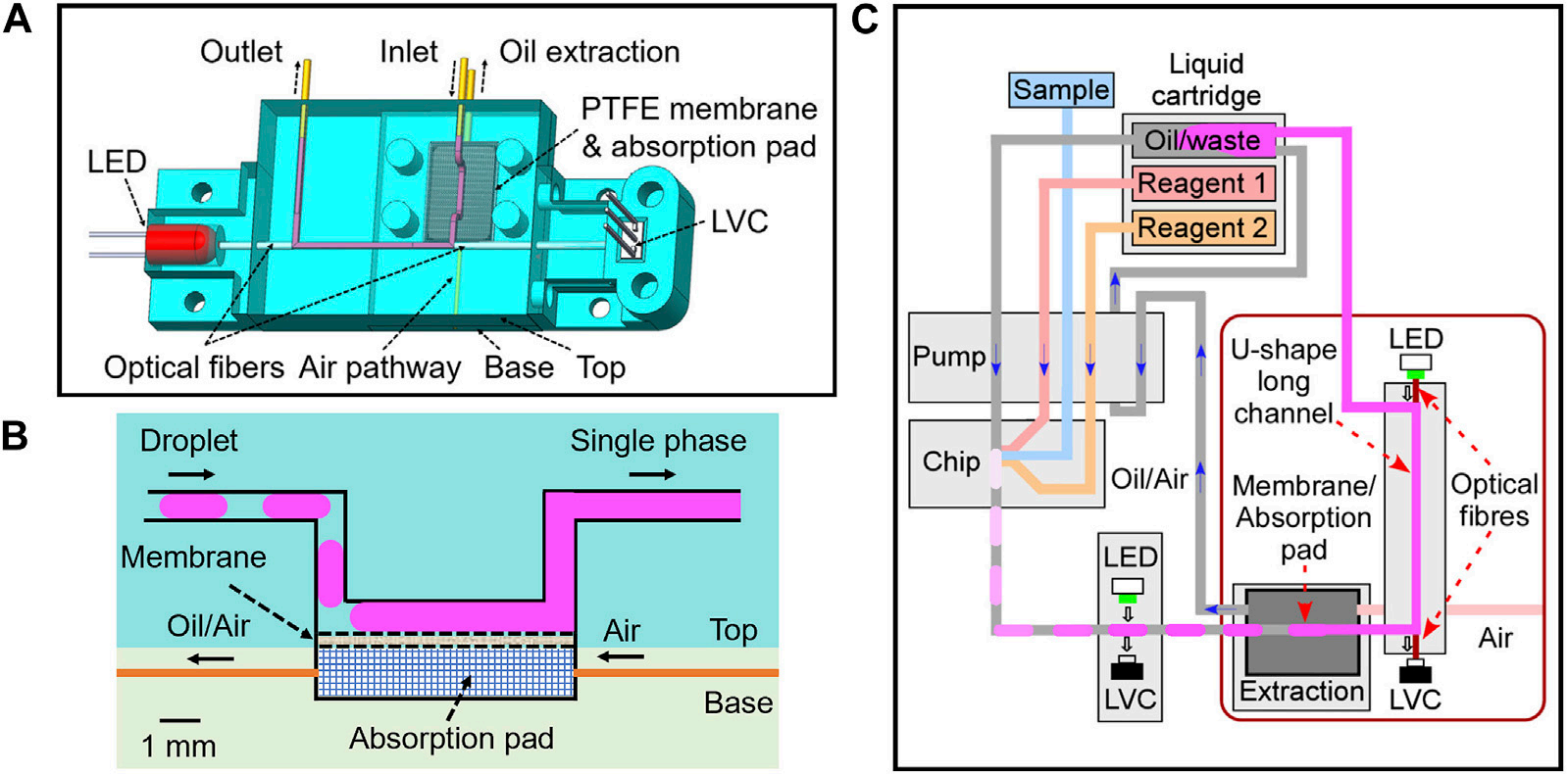
**(A)** 3D schematics for the detection flow cell integrated with LED, LVC, optical fibers and oil extraction unit; **(B)** Cross section view of the open channel with PTFE membrane and absorption pad; **(C)** 2D schematics of fluidic design of the whole system with pump, chip, detection flow cells and liquid cartridge.

Detection flow cells and T-junction droplet microfluidic chips were all 3D printed using an Elegoo Mars 3 Resin printer. The main detection channel featured a square cross-section (0.7 × 0.7 mm). The fabrication and assembly of the long pathlength flow cell (as shown in the 2D and 3D schematics, Figures 1A, B) includes the following steps: 1. Printing a top fluidic part with an open channel and a U-shape channel (Supplementary Figure S1A), and a base part with a groove (Supplementary Figure S1B); 2. Glueing a PTFE membrane (Whatman) to the open channel; 3. Cut two pieces of acrylic optical fibres (outer diameter 0.75 mm, Edmund Optics, United Kingdom) and the ends polished into flat surfaces. The fibres were inserted through the two opening holes of the top part with the end surface in full contact with the channel so that light scattering was minimised; 4. An IR LED (OSRAM, 860 nm) and a light-to-voltage convertor (LVC) (TSL257-LF, RS Components, United Kingdom) were coupled to the optical fibres; 5. An oil absorption pad was fitted into the base chamber; 6. Outlet tubings (PTFE, UT7, Adtech Polymer Engineering Ltd., United Kingdom) and inlet tubings (PEEK, 0.38 mm ID, Cole-Parmer, United Kingdom) glued to the flow cell chip.

The T-junction chip has four inlets and one outlet. A UT7 PTFE tubing was used as the outlet, which was connected to the inlet of the long pathlength flow cell, providing 6 min residence time for the reaction in each droplet. An additional UT7 detection flow cell, as shown in our previous research (Hassan et al., 2018), was used to measure the absorbance of droplets in the UT7 tubing (0.7 mm pathlength) before the droplets entered the oil-removal and long pathlength detection flow cell.

### 2.3 Fluid control

The control the fluids was achieved by our previously reported antiphase peristaltic pumping (Nightingale et al., 2017). Within the microfluidic chip, oil and aqueous phases were introduced into the T-Junction alternatively for droplet generation. The sample-to-reagents volume ratio was precisely controlled at about 3:1:1 via the design of the patterns on the roller of the peristaltic pump. The pumping ratio between the oil introduction and oil extraction was 1:1.1, with the oil extraction flow rate slightly higher than the oil inlet, to avoid the accumulation of oil in the absorption pads. The pump motor, LED and light-to-voltage converter were connected to an interface PCB board using a Teensy 4.1 microcontroller and peripheral components for motor and LED controls. If not specified, the pump ran at a speed of 10 rpm in calibrations resulting in measured flow rates for sample 7.2 μL/min, reagents 2.4 μL/min, oil inlet 3.6 μL/min and oil extraction 3.85 μL/min. In comparison experiments that only use continuous flow (non-droplet), the pump ran at 13 rpm pumping only reagents and sample into the chip, yielding a similar total flow rate of 15.6 μL/min to the droplet flows.

### 2.4 Lab-based analysis using a UV spectrometer

A benchtop UV spectrometer (Lambda35, PerkinElmer, pathlength 10 mm) was used for lab-based analysis of IR dye, phosphate standard solutions and river samples for comparison. The effective optical pathlengths of each microfluidic flow cell were firstly calculated from dividing the absorbance of the standard IR dye solution obtained from the flow cells by the values measured from the spectrometer, and then multiplying the known pathlength (10 mm) of the spectrometer. For the analysis of phosphate standards and river samples, the sample and reagents were first mixed in a 1.5 mL Eppendorf tube for 10 min to perform the PMB reaction before the mixtures were transferred to cuvettes and measured by the spectrometer.

## 3 Results and discussion

### 3.1 The design of the long pathlength flow cell and fluidic system

As shown in Figure 1A, the top part of the flow cell contained a U-shape channel, with inlet and outlet legs on both sides and a straight detection channel in the middle (5–20 mm long for different flow cells). Two pieces of optic fibres were installed on both sides of the detection channel. The other ends of the fibres were connected to an LED on the left and a LVC on the right for absorption measurement.

The inlet channel had a bend with a 5 mm long channel opened to the bottom surface of the top part (Figure 1B). As mentioned in the experimental section, this opening was sealed by a piece of PTFE filter membrane glued to the bottom surface (with the PTFE surface of the membrane facing the channel). The bottom part of the flow cell contained a chamber filled with a cotton wool absorption pad. When the bottom part was fixed to the top part, the absorption pad provided a tight contact and support to the PTFE membrane. The bottom chamber also had two opening holes for active oil extraction and an air pathway as labelled in Figures 1A, B. The detailed fabrication process can be found in the experimental section.

With this design, the PTFE membrane provided an area of hydrophobic porous surface that only allowed the fluorinated oil to pass. Therefore, when the droplets spaced with carrier FC-40 oil were injected into the inlet channel, the oil wetted the membrane and was absorbed into the absorption pad on the other side of the membrane via capillary forces. The aqueous droplets, on the contrary, remained in the channel and merged back to a continuous flow. Note that no surfactant was used in this study. If surfactants were added, a pair of electrodes could be installed close to the channel for droplet merging (Niu et al., 2009).

PTFE surface-assisted oil removal and droplet merging have been demonstrated in other studies for capillary electrophoresis (Niu et al., 2013) and MALDI-MS (Pereira et al., 2013). Here to facilitate continuous oil extraction and even oil reuse for long-term running of the device, we have added an active oil extraction outlet from the chamber as indicated in Figures 1A, B. Figure 1C illustrates the whole system containing both the long pathlength flow cell and other fluidics. The fluidic pumping was carried out by anti-phased peristaltic pump we have designed (Nightingale et al., 2017), with details given in the experimental section. Importantly the pump can accurately control the volume and phase of pumping for each pump line. In this system, we have designed the flow rate in the oil extraction line to be 1.1 times the oil inlet so that no oil build-up in the absorption chamber. The air pathway on the opposite side of the extraction line (as shown in Figure 1A) was added to mitigate the risk of vacuum build-up in the chamber. Droplets, containing either pure standard IR dye solution or the mixture of sample and reagents, were generated at the T-junction of the chip, with an average volume 1.2 μL per droplet and a generation frequency of 10 droplets per minute giving a total flow rate of 15.6 μL/min. Droplet absorbance was first measured by a flow cell with UT7 PTFE tubing (UT7 flow cell) for comparison before droplets entered the long pathlength flow cell.

To calibrate the robustness of oil removal, the system was run continuously for over 4 days. No residual oil in the detection channel was detected or penetration of the aqueous phase through the membrane was observed. Further tests of doubling the total flow rate or reducing the channel opening and corresponding length of PTFE membrane from 5 to 2 mm showed the flow cell worked equally well.

### 3.2 Determination of effective pathlength and Taylor dispersion

**FIGURE 2:**
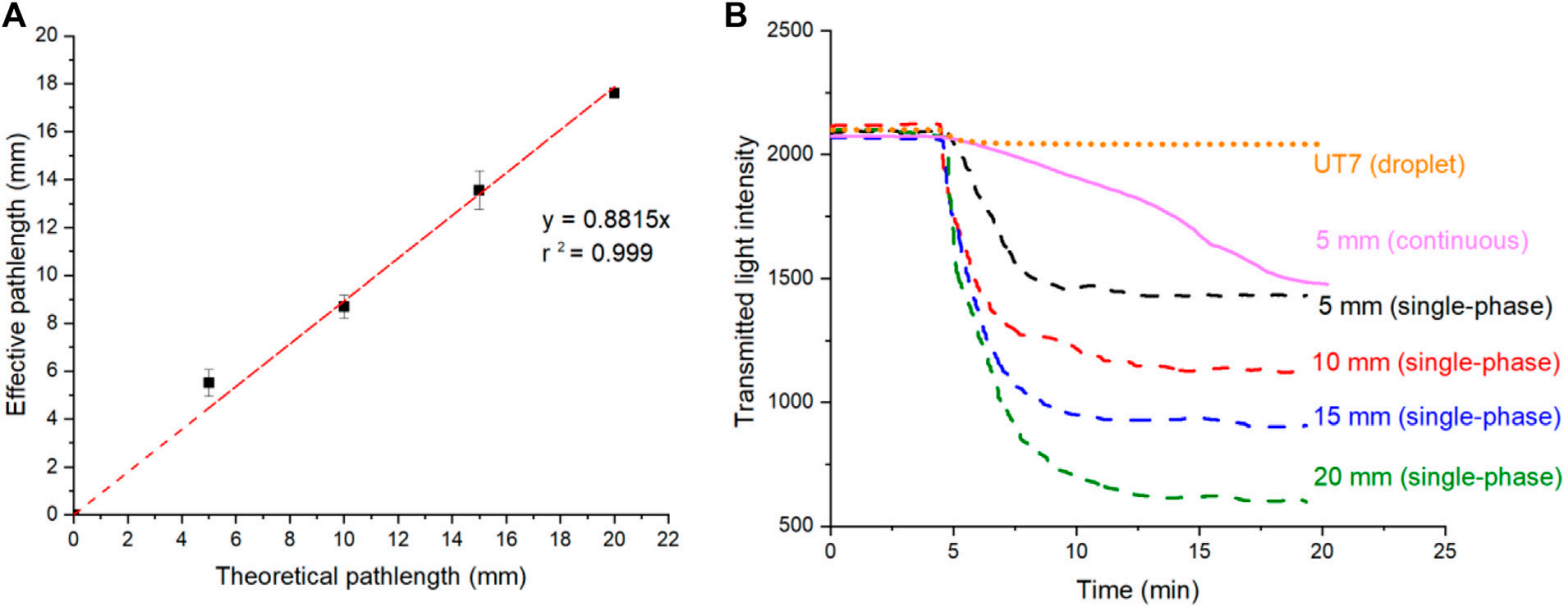
**(A)** Calculated effective optical pathlength under flow cell with different theoretical optical pathlength. The error bars correspond to the standard deviation calculated from three replicates. **(B)** Raw intensity data showing the transition from blank to standard IR dye solution, captured from single-phase flow in long pathlength flow cells (5–20 mm), UT7 flow cell and continuous flow (non-droplet) in 5 mm flow cell.

The effective pathlengths of the designed flow cells were calculated from the absorbance values of manually injected IR dye solution from each flow cell and a conventional UV spectrometer, as described in the experimental section. Figure 2A shows the corresponding effective pathlengths of the flow cells the comparisons with corresponding theoretical pathlengths (or designed U-shape channel lengths). As expected, a linear increase in the effective pathlength was observed as the designed channel extended from 5 to 20 mm. The resulting effective pathlength closely approximated the nominal value, reaching a maximum 18 mm achieved for the flow cell with a 20 mm theoretical channel length. The slight deviations could be attributed to potential errors from the flow cell 3D printing fabrication and measurement errors.

Following the study of effective pathlengths, the flow cells were connected separately to the T-junction chip and fluidic control system, as illustrated in Figure 1C. The measured light intensity from the long pathlength flow cell was recorded simultaneously with that of the UT7 flow cell and plotted as dotted lines in Figure 2B. The figure illustrates the step change transitions from a blank sample to a standard sample containing IR dye. Additionally, a control experiment was conducted by sealing the oil inlet while maintaining the same total flow rate for the three aqueous inlets; these results are represented as a continuous line in Figure 2B. Figure 2B reveals that, following the completion of the transition, the flow cell with a longer pathlength exhibited a lower transmitted light intensity, indicative of higher absorbance. Different transition times were observed from the flow cells. The droplet flow in the UT7 flow cell exhibited the shortest transition time (2 min) due to each droplet being isolated from the channel wall by the carrier oil phase, thereby no Taylor dispersion after droplet generations. Conversely, the transition times for converted single-phase flows ranged from 5 to 8 min as the detection channel length increased from 5 to 20 mm, attributed to the increased fluidic transition time in the detection channels. Nevertheless, these transition times are significantly shorter than that observed in the fully continuous flow of the control experiment, which exceeded 15 min to approach a plateau, even for a 5 mm detection channel. The reduced transition times are advantageous for the fluidic system, enabling the use of lower sample and reagent volumes to achieve accurate readings, minimised the sample-to-signal time, and facilitating the capture of rapidly changing events. These attributes are essential for an online and “near real-time” monitoring system.

The current prototype flow cell was created using an Elegoo Mars 3 resin printer featuring a channel cross-section of 0.7 × 0.7 mm. Much smaller channels could be fabricated using a high-resolution printer or other lithography-based manufacturing techniques to further reduce the transition time and volume required by the detection channels.

### 3.3 Phosphate measurement with the long pathlength flow cell

**FIGURE 3:**
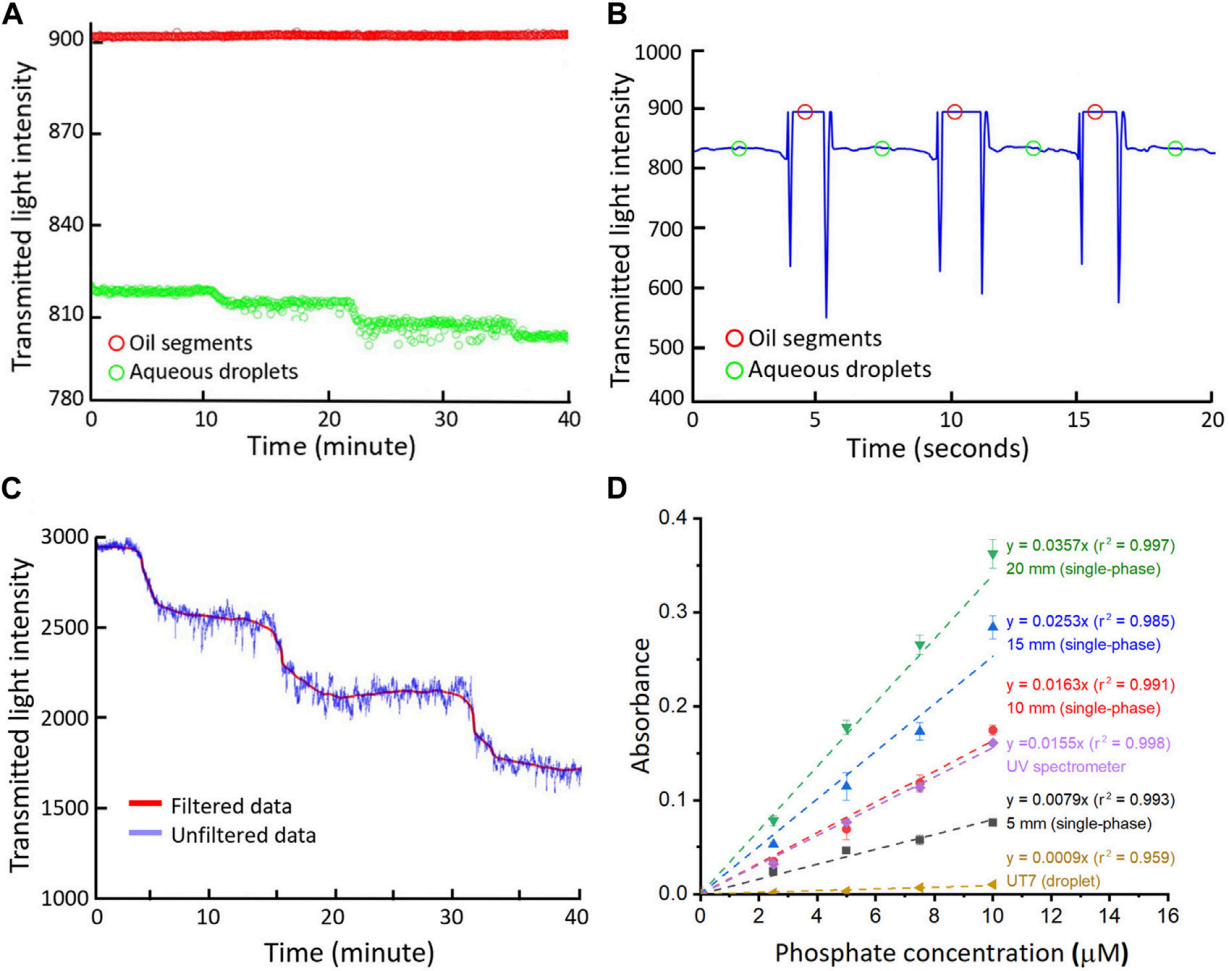
**(A)** Raw intensity data and **(B)** Processed oil segment/aqueous droplet intensity from the measurement of phosphate standards in UT7 flow cell; **(C)** Filtered and raw intensity date from the measurement of phosphate standards in 20 mm long pathlength flow cell; **(D)** Calibration graphs for phosphate assay in different flow cells, number given are nominal designed pathlengths. The error bars correspond to the standard deviation calculated from three replicates.

Followed by the characterisation of the designed flow cells, we further used them in conjunction with the droplet-based PMB assay to measure orthophosphate (PO_4_^3−^) in standard solutions. Phosphate samples were mixed with PMB reagents for reaction within droplets and then merged into a single-phase flow after oil extraction, allowing for long pathlength detection in the U-shape channels. Figures 3A, B illustrate the transmitted light intensity data collected from the UT7 flow cell installed prior to the oil-removal and the long pathlength flow cell (Figure 2C). Here, aqueous droplets stand for the droplets produced from reagents and standard phosphate solutions, which were segmented by the oil in the UT7 PTFE tubing. Incremental changes in intensity can be seen as we increased the phosphate concentrations in the standard solutions from 2.5 to 7.5 μM as shown in Figure 3A, where each point represents the plateau of the oil or aqueous sample droplet as detailed in Figure 3B. Similarly, the 20 mm flow cell showed a continuous stepwise change in the intensity (Figure 3C) as the phosphate concentration increased. The full calibration graphs for long pathlength flow cells (5–20 mm), a UT7 flow cell and a conventional UV spectrometer are illustrated in Figure 3D. All measurements showed good linearity with R-square values close to 1, and steeper slopes/gradients as the pathlengths were increased. The data from 10 mm flow cell overlapped largely with that of the UV spectrometer. The slight differences are potentially due to different reaction times or fabrication errors. Using 3-sigma method (Loock and Wentzell, 2012), the resulting limit of detections can be calculated as 3.87 μM (for a UT7 flow cell), 2.83 μM (5 mm), 1.37 μM (10 mm), 0.89 μM (15 mm) and 0.66 μM (20 mm).

### 3.4 River sample analysis using a 20 mm long pathlength flow cell

**FIGURE 4:**
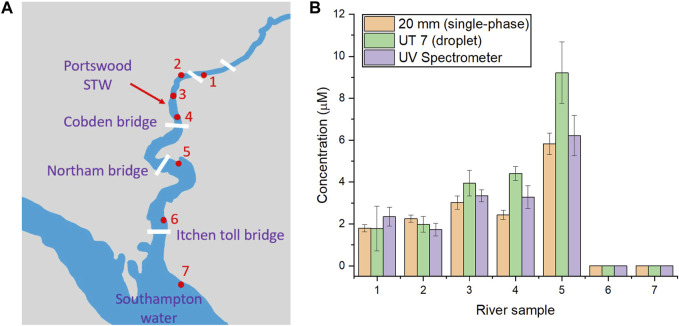
**(A)** Map illustration of sample collection points; **(B)** The chart for measured phosphate concentrations at different collection points around River Itchen (each measurement was repeated for three times). The error bars correspond to the standard deviation calculated from three replicates.

Since the 20 mm flow cell presents the highest sensitivity with the lowest limit of detection (0.66 μM), it was selected for the quantification of river samples normally containing low concentrations of phosphate. The samples were collected from different locations along the river Itchen, United Kingdom, as shown in Figure 4A, with a method mentioned in the experimental section. Similar to the calibration of the standard solutions in Section 3.3, the river sample was pumped into the T-junction chip and mixed with the two reagents in the droplet for PMB assay. The corresponding phosphate levels in each sample were calculated from the absorbance values measured from UT7 and 20 mm flow cell using calibrations established in Figure 3D, as plotted in the bar chat in Figure 4B, alongside the data obtained from benchtop spectrometer analysis. Notably, phosphate levels varied across different locations along the river. At the limit of the tidal range (sites 1–2), the concentrations were around 2 μM. Moving downstream to sites 3 and 4, the phosphate level increased to around 3 μM, reaching 6 μM near Northam Bridge (site 5). Further downstream to point 6 and 7, where the river met Southampton Water below Itchen Bridge, the phosphate level became undetectable. This increase in phosphate were previously observed (Veeck, 2007) and was linked to an anthropogenic input, specifically from a small sewage treatment works located at Portswood near sampling site 3. As the tide went down, phosphate could migrate to site 5 but diluted upon reaching Southampton Water at Site 6 and 7. Overall, the results from the long pathlength flow cell aligned with the data obtained from UV spectrometer, providing less error in measurement compared to that of the UT7 flow cell. In addition to single point measurement from collected river samples, this highly-sensitive flow cell is promising to be integrated into an *in situ* device, similar to our previously reported water/soil sensors (Nightingale et al., 2019a; Lu et al., 2024), for continuous monitoring of phosphate in natural water.

## 4 Conclusion

We have successfully developed a robust oil removal and long pathlength flow cell for UV-Vis absorption detection of droplet microfluidics. A PTFE membrane was used to effectively remove the oil with droplets merging into a continuous flow before detection in the extended U-shaped channel. The continuous oil removal by the pump ensures the system can run continuously without the change of the absorption pad. This “hybrid” approach combines the advantages of rapid mixing, minimised Taylor dispersion from droplet microfluidics and the sensitive detection offered by single-phase long pathlength channel, therefore providing a practical solution for using droplet microfluidics to measure and monitor trace elements in liquids using the well-established colorimetric assays. Phosphate measurement from river water samples was used as a proof-of-principle application of this flow cell, showing reliable measurement and consistency with that of the gold-standard method (UV spectrometer). The flow cell has the potential to be used for *in situ* and continuous monitoring of other important nutrients or pollutants [e.g., nitrate, ammonium (Choosang et al., 2018)] in natural aquatic environment or wastewater treatment. In addition, we envisage the flow cell can be applied to wearable or point-of-care biomedical sensors for real-time monitoring of chemical biomarkers via existing colorimetric assays, such as biomolecules [e.g., glucose, lactate (Nightingale et al., 2019b)], drugs with narrow therapeutic windows [e.g., lithium carbonate (Komatsu et al., 2020)], and large proteins with physical transition behaviours (Toprakcioglu et al., 2019).

## Data Availability

The raw data supporting the conclusion of this article will be made available by the authors, without undue reservation.
