# Supplementary figures and images for: Highly sensitive absorbance measurement using droplet microfluidics integrated with an oil extraction and long pathlength detection flow cell
Source: Front Chem. 2024 May 13;12:1394388. doi: 10.3389/fchem.2024.1394388 (PMC11129082; doi:10.3389/fchem.2024.1394388)

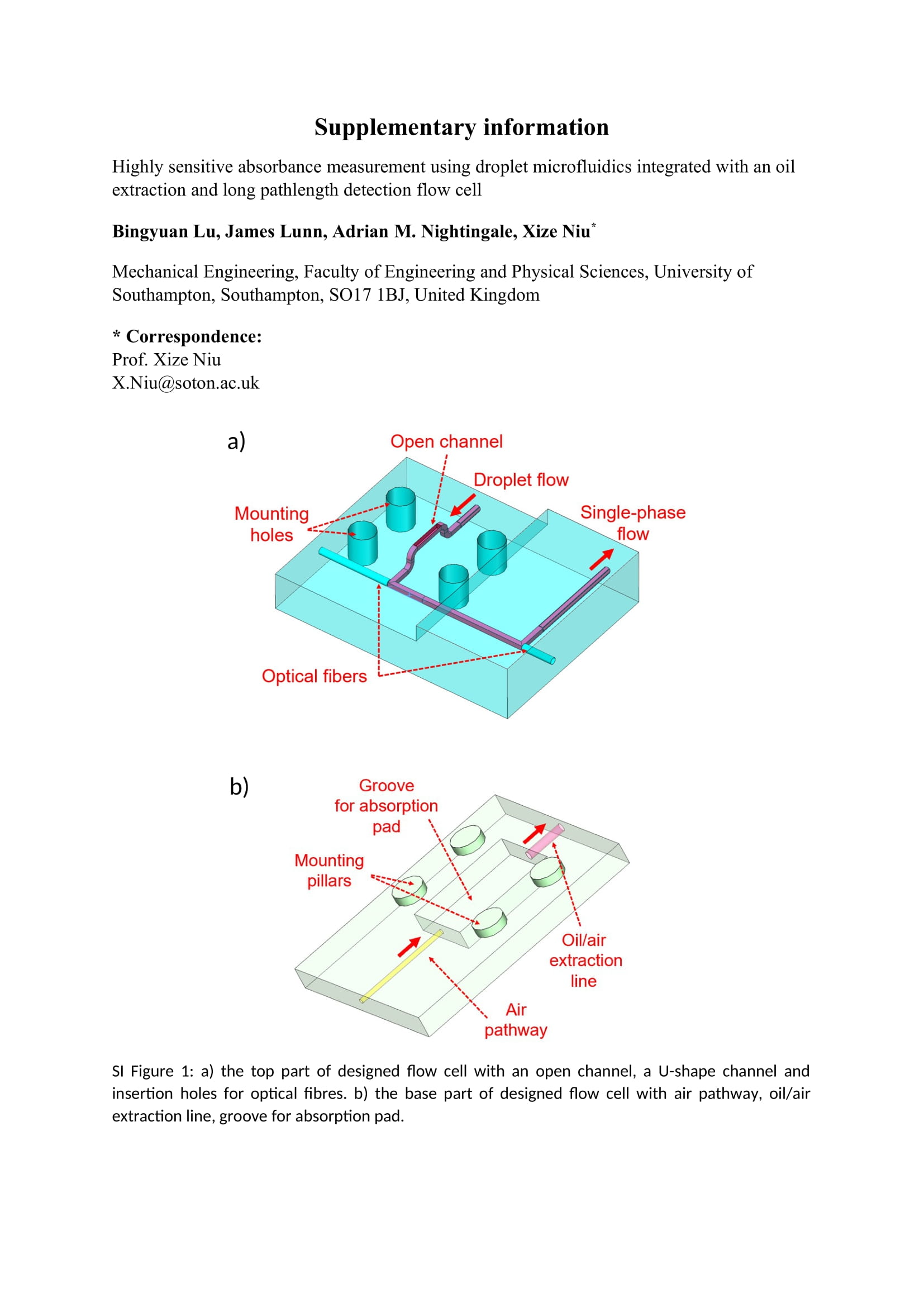

Supplement: Supplementary file 1 [file Image1.jpg]
